# Characterization and selection of probiotic lactic acid bacteria from different dietary sources for development of functional foods

**DOI:** 10.3389/fmicb.2023.1170725

**Published:** 2023-05-05

**Authors:** Khushboo, Arun Karnwal, Tabarak Malik

**Affiliations:** ^1^Department of Microbiology, School of Bioengineering and Biosciences, Lovely Professional University, Phagwara, India; ^2^Department of Biomedical Sciences, Jimma University, Jimma, Ethiopia

**Keywords:** *Lactobacillus delbrueckii*, *Lactobacillus acidophilus*, millet, sugar utilization, curd, pickle

## Abstract

**Introduction:**

Dietary sources have an abundance of bacteria, mainly lactic acid bacteria (LABs), which have long been regarded as probiotics in humans and animals. Lactic acid bacteria (LAB) have been used as probiotic agents due to their ability to produce a variety of beneficial compounds for cultivars and their status as safe microorganisms.

**Methods:**

In this current study, the lactic acid bacteria (LAB) were isolated from several dietary sources such as curd, pickle, milk, and wheat dough. The principal focus of this study was to determine the survivability of these microorganisms in the gastrointestinal tract and to use promising strains to create probiotic drinks with numerous health benefits. The isolates were identified using a combination of morphological, biochemical, molecular and sugar fermentation patterns, like phenotypic characteristics, sugar fermentation, MR-VP reaction, catalase test, urease test, oxidase test, H_2_S production, NH_3_ production synthesis from arginine, citrate utilization, indole test, and 16s rRNA sequencing.

**Results:**

Two (CM1 and OS1) of the 60 isolates obtained showed the best probiotic results and were identified as Lactobacillus acidophilus CM1 and *Lactobacillus delbrueckii* OS1. These organism sequences were submitted to Gen bank with accession numbers OP811266.1 and OP824643.1, respectively. The acid tolerance test results indicated that most strains could survive significantly in an acidic environment with pH levels of 2 and 3. Similarly, the salt tolerance test results showed that both *Lactobacillus acidophilus* CM1 and *Lactobacillus delbrueckii* OS1 could survive at 4 and 6% NaCl levels significantly. The isolates also showed their ability to ferment sugars such as lactose xylose, glucose, sucrose, and fructose.

**Discussion:**

In conclusion, the study showed that the bacteria isolated from different food sources were indeed probiotic lactic acid bacteria and had probiotic properties. These isolates hold potential for future research in the formulation of millet-based probiotic beverages. However, further studies are required to confirm their effectiveness and safety in improving human health. This research provides a foundation for developing functional foods and drinks that can positively affect human health by incorporating probiotic microorganism.

## 1. Introduction

Probiotics are living microorganisms, typically bacteria that provide health benefits to the host when administered in suitable numbers (Al-Dhabi et al., 2020; Zhang et al., 2020). They are commonly found in fermented foods like yogurt, kefir, sauerkraut, and kimchi and are also available as supplements. The most common bacterial genera found in commercially available probiotic supplements are *Lactobacillus, Bifidobacterium, Streptococcus*, and *Enterococcus* (Selvaraj and Gurumurthy, 2023; Wang et al., 2023). Lactic acid bacteria (LAB) are a significant type of bacteria that are widely utilized in the food, dairy, probiotic, and beverage manufacturing industries. They are Generally Regarded as Safe (GRAS) and possess unique features that make them ideal for these applications (Zhang et al., 2020; Khusro et al., 2021). LAB, particularly lactobacillus, is widely used in the food industry to produce primary or starter cultures for various dairy products. LAB has gained significant attention recently due to its ability to modulate the human host system against foodborne pathogens. Thus, these bacteria are currently being explored for their potential use as a bio-preservative agent in the food and dairy industries and an antibiotic alternative in human medical treatments (Lashani et al., 2020; Salim et al., 2020; Rashed et al., 2022). Probiotics can cure various health conditions, such as inflammatory bowel disease, irritable bowel syndrome, constipation, antibiotic-associated diarrhea, acute diarrhea, allergy-related diseases, hypertension, and diabetes. However, for probiotic strains to function appropriately, they must exhibit certain beneficial qualities such as tolerance to gastrointestinal illnesses, adhesion to epithelial cells, ability to absorb cholesterol, hydrolysis of bile salts, safety against virulence genes, non-hemolytic activity, antibiotic sensitivity, antibacterial properties, and viability during fermentation and storage processes (A'inurrofiqin et al., 2022; M'Hamed et al., 2022). Moreover, it is preferable for probiotic strains to have industrial important characteristics like tolerance to heat treatment, mainly spray drying, and exopolysaccharide (EPS) production, which can benefit consumers as non-digestible fiber or food flavor. Probiotic strains can also make fermented functional foods with more significant positive health effects than traditional food products. Researchers continuously search for novel strains of LAB bacteria that exhibit superior probiotic properties in various foods (Selvaraj and Gurumurthy, 2023). Despite the potential benefits of probiotics, caution must be taken when choosing a probiotic supplement, as not all strains have proven effective. It is also important to note that probiotics are not a substitute for a healthy diet and lifestyle but can be used to complement them.

Probiotic supplements have gained much attention in recent years due to their potential to reduce the likelihood of infectious infections and the need for antibiotics. This is a significant development, as antibiotic treatment is often unsuccessful due to the development of drug resistance (Das et al., 2022), which can make infection a constant source of worry for those who have a history of suffering from recurrent infections. Untreated infections can lead to significant illness and hospitalization, making probiotics an increasingly attractive option for preventing or delaying the formation of bacteria resistant to multiple drugs. Probiotics have been found to significantly impact the fight against infectious diseases through their effects on the epithelium, the synthesis of antimicrobial substances, and competitive exclusion (Lim and Im, 2009; Kirtzalidou et al., 2011). Le and Yang (2022) and Le et al. (2023) suggested that analyzing the metabolic pathways of probiotic bacteria can explain their metabolic profile and provide guidance for industrial production of functional fermented milk. Recent research has focused on using millet and beverages prepared from millet as appropriate ingredients for probiotic products (Desrouilleres et al., 2020). These products effectively promote the growth of probiotic bacteria and include a variety of beneficial nutritional components such as dietary fiber, vitamins, and minerals. Millet-based drinks, including finger and pearl millet, are effective with other probiotic strains (Sachdev et al., 2023). Grains, millets, and legumes are excellent sources of nutritional components, such as fibers that function as prebiotics and probiotic food. They also shield probiotic cells from harmful gastrointestinal conditions, making them ideal substrates for developing probiotic products (Desrouilleres et al., 2020). These food sources provide essential elements such as proteins, carbohydrates, vitamins B and E, iron, trace minerals, and fiber. Consuming cereal is also associated with a reduced chance of acquiring a variety of chronic illnesses, making it an even more attractive food choice (Bembem and Agrahar-Murugkar, 2020). Therefore, the present research aimed to isolate, characterize, and identify Lactobacillus isolates from the oral cavity and different dietary sources to explore their probiotics properties (KIA assay, non-hemolytic assay, Acid tolerance, Bile tolerance, NaCl tolerance, and antimicrobial activity against pathogens) and the possibility of using these isolated LABs as the potential probiotic organisms for millet-based probiotic products production.

## 2. Materials and methods

### 2.1. Sample collection

The study aimed to isolate strains of lactic acid bacteria (LAB) from fermented and non-fermented foods such as pickle, milk, curd, and wheat dough. In order to achieve this, samples were collected from various regions, specifically Jalandhar, Amritsar, and Mukatsar in Punjab, as well as Kanina Khas and Maherdragarh in Haryana (Table 1). These locations were chosen because of their unique food cultures, which could potentially contribute to the diversity of LAB strains present in the samples. The samples were carefully collected and transported to the laboratory for further analysis as described by Zhang et al. (2022).

**Table 1:** Location details of food samples used for the isolation of probiotic organisms.

**Place**	**Latitude**	**Longitude**
Jalandhar region of Punjab	31.326060	75.575620
Amritsar region of Punjab	31.639770	74.838760
Mukatsar region of Punjab	30.238810	75.189790
Kanina Khas region of Haryana	28.328760	76.306640
Maherdragarh region of Haryana	28.329923	70.305740

### 2.2. Isolation, purification, and screening of lactic acid bacteria

Microbiologic methods were employed to process the samples, involving streaking them onto MRS agar (de Man, Rogosa, and Sharpe) from HiMedia, India, and incubating them in anaerobic conditions at 37°C for 24–48 h. The LAB colonies that exhibited typical characteristics were hand-picked, sub-cultured, and grown in MRS broth. The colonies were assessed based on morphological characteristics, including Gram staining, colony morphology, motility test, endospore test; biochemical tests such as MR-VP Test, Indole Test, Citrate Utilization Test, Nitrate reduction test and gas production test, oxidase, catalase test, and fermentation patterns of different sugars, as well as morphological traits such as colony color, shape, size, elevation, and density. The results were then compared to the Bergey's Manual of determinative Bacteriology (Holt et al., 1994) for further analysis. The most promising isolates, which showed growth during sub-culturing, were selected for further investigation. The bacterial culture was then preserved in MRS agar slant and stored at 4°C for future studies.

### 2.3. Characterization of isolates for probiotic properties

#### 2.3.1. KIA test

All the purified isolates were subjected to Kliger's Iron Agar (KIA) test (Sefcova et al., 2021) to determine their mechanism of lactose and glucose consumption. A freshly prepared culture was inoculated by streaking the slant and stabbing the butt. The results were recorded after incubation at 37°C for 24 h. The test results showed the color changes of the slant and butt, as well as the generation of H_2_S gas or other gases (Hatami et al., 2022). If only glucose was fermented, the slant was acidic and the butt was alkaline. If both lactose and glucose were fermented, both the butt and slant were acidic, and if neither lactose nor glucose was fermented, both the butt and slant were alkaline. The medium could become blackened due to the formation of hydrogen sulfide and gas production, which would lead to the formation of bubbles in the tube (Bazireh et al., 2020).

#### 2.3.2. Casein hydrolysis activity

The protease activity was determined using an MRS agar plate with a 1% skim milk solution. The bacterial cultures were inoculated onto the plate and incubated at 37°C for 24–48 h. The presence of clear zones around the cultures indicated the presence of protease activity (Chandok et al., 2014).

#### 2.3.3. Hemolytic activity

Using a blood agar base with 5% (w/v) sheep blood, the isolates' hemolytic activity was determined. The plates were incubated at 37°C for 48 h, after which the isolated cultures' hemolytic activity was analyzed and classified according to how much red blood cell lysis there was in the medium around the colonies. The presence of clear zones around colonies indicated α-hemolysis, the presence of green zones around colonies all suggested β-hemolysis and the absence of any zones surrounding colonies on the blood agar plates indicated γ-hemolysis. Strains were deemed safe only if they displayed–hemolysis (Asadi et al., 2022; Wei et al., 2022).

#### 2.3.4. Arginine hydrolysis

The process of determining the presence of arginine dihydrolase enzyme involves aseptically transferring a pure culture's inoculum to a sterile tube filled with arginine dihydrolase broth. The inoculated tube is then incubated for 24 h at 37°C to gather preliminary results. For the pH to be lowered, the microorganism must first consume the available glucose, as described by Soccol et al. (2010), which is indicated by a change in color from purple to yellow. The arginine dihydrolase enzyme is activated when the medium becomes more acidic. The culture is maintained at 37°C for another 24 h to allow the microbe sufficient time to utilize the arginine. After 48 h of incubation, the tube is observed to obtain the final results. A return in color from yellow to purple signifies a positive result for the arginine dihydrolase test.

### 2.4. Study the probiotic attributes of microorganisms

#### 2.4.1. Acid tolerance test

MRS broth was adjusted with 1N HCl, and 1N NaOH at pH levels 1, 2, 3, and 4, and freshly prepared bacterial cultures were added to the respective MRS broth in test tubes. The tubes were incubated for 48 h at 37°C, and the turbidity of the culture media was monitored after 24–48 h. The negative control, which was only the media, showed no growth (Chen et al., 2022).

#### 2.4.2. NaCl tolerance test

The NaCl tolerance of isolated bacterial cultures was investigated using MRS broth with the NaCl concentration of 2, 4, 6, and 8%. A freshly prepared culture was incubated for 48 h at 37°C, and the turbidity was measured after 24 and 48 h. The negative control, which was only the media, showed no growth (Chen et al., 2022).

#### 2.4.3. Bile tolerance test

The tolerance to the bile salt of the isolates was evaluated by using 10 mL of MRS broth supplemented with 0.5, 1.0, 1.5, and 2% bile salt. The culture was incubated at 37°C, and samples were taken at 24 h. The negative control, which was only the media, showed no growth (Chen et al., 2022).

#### 2.4.4. Antimicrobial activity

The antimicrobial activity of the isolates was tested using culture-free supernatants against the indicator *bacteria Bacillus cereus, Staphylococcus aureus, Salmonella typhimurium Escherichia coli*, and *Enterococcus faecalis*. The activity was measured using the standard agar well diffusion technique on Mueller Hinton Agar (MHA) plates. To counteract the inhibitory effects of lactic acid, the pH of all supernatants was adjusted to 6.5. The zone of inhibition was measured in mm after 24 h of incubation at 37°C (Chen et al., 2022).

### 2.5. Molecular characterization

The identification of LAB isolates was determined through a combination of morphological and phenotypic characterizations, including both biochemical and physiological traits. The process of 16S rDNA sequencing was utilized to further confirm the identity of the isolates. The isolates were sent to Yaazh Xenomics in Coimbatore, Tamilnadu, India for sequencing. The genomic DNA was extracted using a chloroform-isoamyl alcohol method and amplified via PCR. The amplification procedures were performed using the EXpure Microbial DNA Isolation Kit from Bogar Bio Bee Shops Pvt Ltd. (Swain et al., 2022). The universal primers used for the 16S rDNA were 27F: 5' AGA GTT TGA TCC TGG CTC AG 3' and 1492R: 5' ACG GCT ACC TTG TTA CGA CTT 3'.

The amplified PCR product was analyzed using ethidium bromide staining on a 1.2% (w/v) agarose gel (HiMedia, India). The purified PCR product, which included both forward and reverse sequences, was then sequenced. The sequence was compared to the NCBI nucleotide database using the BLAST software. Once uploaded to GenBank, nucleotide BLAST was performed. The phylogenetic tree was constructed using the Jukes-Cantor corrected distance model. The sequences of CM1 and OS1 have been deposited in the Genbank database.

### 2.6. Statistical

The data were analyzed using SPSS version 20 (SPSS Inc., Chicago, IL, USA) and presented as mean ± standard deviation (SD) based on three independent experiments conducted in triplicate. One-way ANOVA followed by Tukey's *post-hoc* test was employed for statistical comparison analysis. Data with *p* < 0.05 were considered statistically significant.

## 3. Result

Probiotics can be found in fermented and non-fermented foods, which primarily include several *Lactobacilli* species. The most popular foods that contain probiotics is milk, curd, pickle etc. In this study 50 samples were collected from Different food sources including cow milk, buffalo milk, curd, pickle and wheat dough (refined) and 10 samples were collected from the oral cavity of human. MRS agar media (Pumriw et al., 2021) was utilized to isolate bacteria and incubated for 48 h at 37°C. The resulting colonies were labeled with the name of the sample and a serial number, such as 1, 2, 3, and so on as presented in Table 2. Sixty colonies, with different morphological properties, isolated on MRS agar from oral cavity and various foods (Table 2) were Gram-stained and tested for catalase.

**Table 2:** Various types of samples utilized for the isolation of probiotic bacteria, along with the number of isolates that were assessed for their probiotic characteristics.

**Sample type**	**Abbreviation**	**Bacterial isolates**
Oral cavity	OS	OS1 to OS10
Curd	C	C1 to C10
Cow milk	CM	CM1 to CM10
Buffalo milk	BM	BM1 to BM10
Pickle	PK	PK1 to PK10
fermented dough	FB	FB1 to FB10

Twenty three isolates were Gram-positive, cocci shaped, and catalase-positive whereas 21 isolates were Gram-positive, rod shaped, and catalase-positive. Sixteen isolates (CM1, CM2, CM6, CM9, BM1, BM2, BM3, C2, C3, C4, OS1, OS6, PK5, PK6, FB4, and FB7) were Gram-positive, rod shaped, and catalase-negative; these isolates were subjected to further examination. All 60 isolates were non-motile, non-endospore former and showed strong growth at 37°C in MRS media under anaerobic conditions. Subsequently, the isolated purified 16 bacterial colonies (Gram-positive, rods, catalase negative) underwent further identification using microscopic, macroscopic and biochemical methods as described in Bergey's manual of determinative bacteriology (Holt et al., 1994).

### 3.1. Characterization and screening for probiotic properties of bacterial isolates

In order to identify the prevalence of Lactic Acid Bacteria (LAB), the researchers conducted a series of biochemical assays on 16 isolates, as described by Taye et al. (2021). These assays included MR, VP, H_2_S, indole production, acid/gas production from glucose, ammonia production, nitrate reduction, and citrate utilization. These tests aimed to identify the isolates based on their biochemical characteristics preliminarily. Among the 16 isolates, only three (CM6, BM2, and OS6) were MR negative, indicating that they did not produce mixed acid fermentation products from glucose. In addition, only one isolate (OS1) was found to be citrate-positive, indicating that it could utilize citrate as a carbon source. All remaining isolates were negative for MR, VP, H_2_S, Indole, nitrate, and citrate utilization, suggesting that they may be different LAB strains.

The KIA test results revealed distinct variations in the types of bacteria found in the isolates (Sefcova et al., 2021). All strains tested positive for the KIA test, indicating their ability to ferment sugars. Out of the 16 isolates, 10 (CM1, CM2, CM6, CM9, BM2, BM3, OS1, OS6, FB4, and FB6) displayed Alkaline Slant/Alkaline Butt characteristics, one (PK5) was positive for Alkaline Slant/Acid Butt, and five exhibited Acid Slant/Acid Butt characteristics (BM1, C2, C3, C4, and PK6) during the KIA assay as shown in Table 3. The Alkaline Slant/Alkaline Butt test indicated the presence of non-fermentative gram-positive bacteria that could not produce acids through glucose or lactose fermentation, as there was no change in color and no fermentation of carbohydrates. The Alkaline Slant/Acid Butt test revealed that the bacteria were non-lactose fermenters but could ferment glucose. This is a characteristic of bacteria that cannot digest lactose (Sefcova et al., 2021). Initially, the medium became more acidic due to glucose fermentation. However, it returned to an alkaline pH due to the production of alkaline amines near the surface through oxidative decarboxylation of peptides derived from the proteins in the medium in the presence of oxygen. In contrast, the Acid Slant/Acid Butt test showed that lactose-fermenting bacteria could completely and permanently acidify the slant and depth of the tube, indicating their ability to ferment glucose and lactose. None of the isolates displayed any hemolytic activity, ensuring they were safe for human consumption. The isolates exhibited yellow during the arginine hydrolysis test, indicating their inability to convert arginine to ammonia. This could be because they could not use arginine, an amino acid, as a carbon and energy source.

**Table 3:** Evaluation of bacterial isolates for probiotic properties.

**Bacterial isolates**	**KIA**	**Casein hydrolysis**	**Hemolytic activity**	**Arginine hydrolysis**
CM1	Alkaline/alkaline	_+_	Gamma	+
CM2	Alkaline/alkaline	+	Gamma	+
CM6	Alkaline/alkaline	+	Gamma	+
CM9	Alkaline/alkaline	+	Gamma	+
BM1	Acid/acid	+	Gamma	+
BM2	Alkaline/alkaline	+	Gamma	+
BM3	Alkaline/alkaline	+	Gamma	+
C2	Acid/acid	+	Gamma	+
C3	Acid/acid	+	Gamma	+
C4	Acid/acid	+	Gamma	+
OS1	Alkaline/alkaline	+	Gamma	+
OS6	Alkaline/alkaline	+	Gamma	+
PK5	Alkaline/Acid	+	Gamma	+
PK6	Acid/acid	+	Gamma	+
FB4	Alkaline/alkaline	+	Gamma	+
FB7	Alkaline/alkaline	+	Gamma	+

### 3.2. Carbohydrate fermentation

The study investigated the sugar utilization patterns of 11 different sugars by lactic acid bacteria isolates. The results indicated that the tested sugars could be broadly categorized into two groups based on the percentage of strains that could utilize them effectively. The majority of the strains were capable of fermenting sugars such as Glucose (91.63%), Lactose (83.3%), and Maltose and Fructose (66.64%). On the other hand, Mannose (58.31%), Galactose (49.98%), Mannitol, Ribose, and Sucrose (41.65%), Starch (16.66%), and Arabinose (8.33%) were found to be less utilized by the isolates, indicating limited use of these sugars in further studies. The sugar utilization pattern varied among the different strains, highlighting the unique characteristics of each strain. Based on their specific sugar utilization patterns and other desirable characteristics, the best lactic acid bacteria can be selected as potential probiotic candidates for further investigation. Overall, these findings provide valuable insights into the sugar utilization capabilities of lactic acid bacteria and their potential as probiotic agents (Lim and Im, 2009).

### 3.3. Study the probiotic attributes of microorganisms

**Figure 1:**
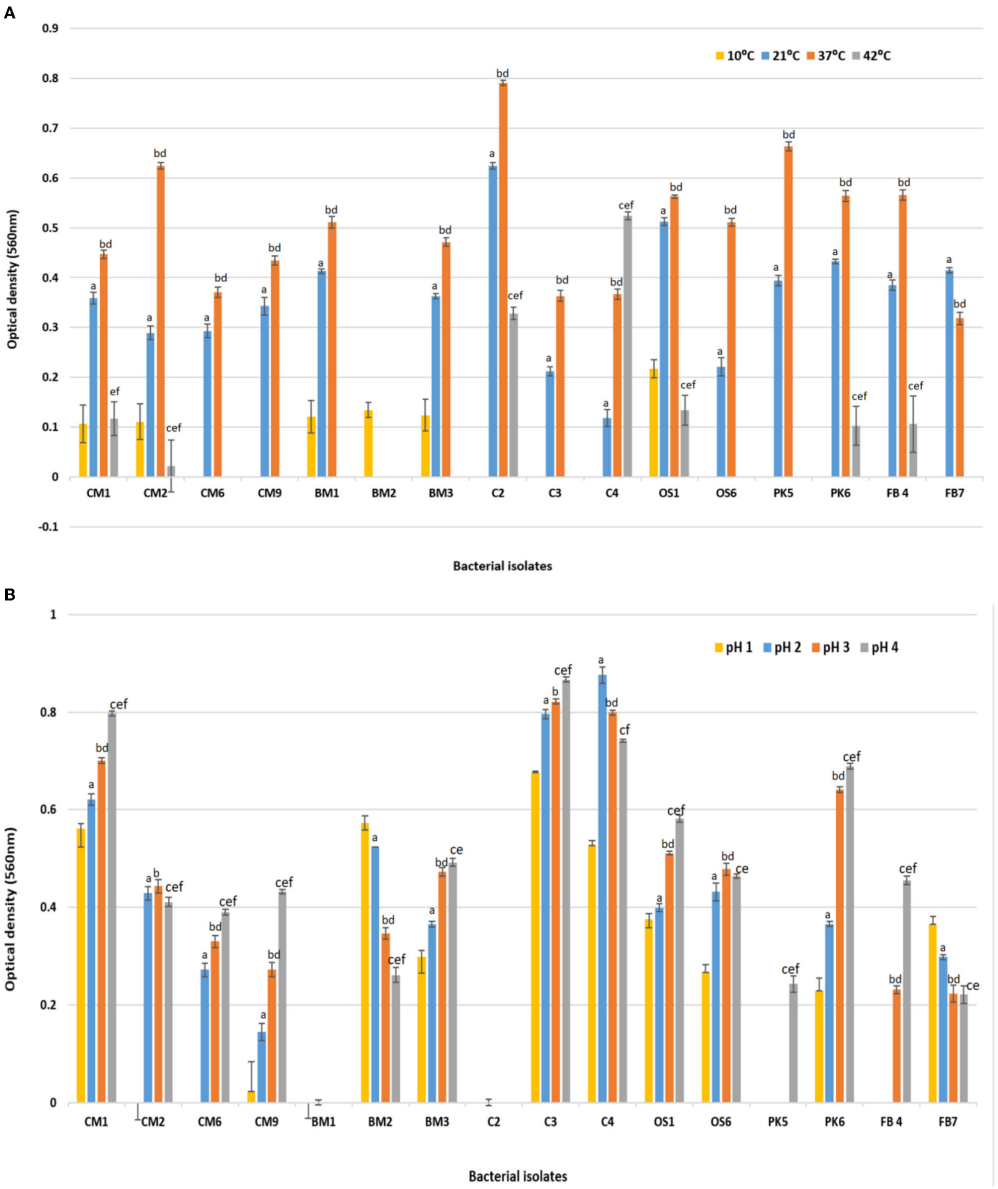
Effect of **(A)** temperature 10, 21, 37, and 42°C on the survival rate or growth of bacterial isolates after 24 h of incubation. Superscript small case alphabets presented in chart bars are presenting the significant difference at *p* < 0.05 between trials (temperature 10–21°C: a, 10–37°C: b, 10–42°C: c, 21–37°C: d, 21–42°C: e, 37–42°C: f). **(B)** Effect of acidic pH (1, 2, 3, and 4) on the survival rate or growth of bacterial isolates after 180 min of incubation at 37°C. Superscript small case alphabets presented in chart bars are presenting the significant difference at *p* < 0.05 between trials (pH 1–2: a, 1–3: b, 1–4: c, 2–3: d, 2–4: e, 3–4: f). *All data were expressed as mean ± SD.

The probiotic properties of all 16 isolates were evaluated, including their ability to tolerate varying levels of temperature change, pH change, NaCl concentration, Bile salt tolerance, and antimicrobial properties (Dabiré et al., 2022; M'Hamed et al., 2022). Figure 1A displays the temperature study results, where the ability of each isolate to grow in MRS broth at 10, 21, 37, and 42°C was tested. Growth was observed in 37.2% of isolates at 10°C, while 43.8% of isolates were able to grow at 42°C and shown a significant reduction in growth compared to 37°C as presented in Figure 1A. The majority of isolates (93.7%) exhibited significantly better growth at both 21 and 37°C, with the highest growth rate observed at 37°C (which is equivalent to human body temperature) compared to 10 and 42°C. Values were means of three replicates, dissimilar letters show significant difference (*p* < 0.05).

Figure 1B also presents the acid tolerance results of the various bacterial isolates that were exposed to different levels of acidic pH (1, 2, 3, and 4) at 37°C for 24 h, with pH 7.0 serving as the control. Isolates CM1, CM9, BM2, BM3, C3, C4, OS1, OS6, PK6, and FB7 survived at pH levels 1, 2, 3, and 4, albeit with a decrease in cell concentration compared to pH 7. The growth of bacteria after incubation in varying pH (2, 3, and 4) were significantly different compared with the growth at 1 pH (*p* < 0.05). Among the isolates, CM1 and OS1 were able to survive in acidic conditions for 3 h, whereas the remaining isolates showed a lower survival rate and could not tolerate the low pH for more than 90 min. Most isolates displayed significantly better survival at pH 3 compared to pH 1 and 2, although there was still a decrease in cell concentration compared to control pH 7. Values were means of three replicates, dissimilar letters show significant difference (*p* < 0.05).

**Figure 2:**
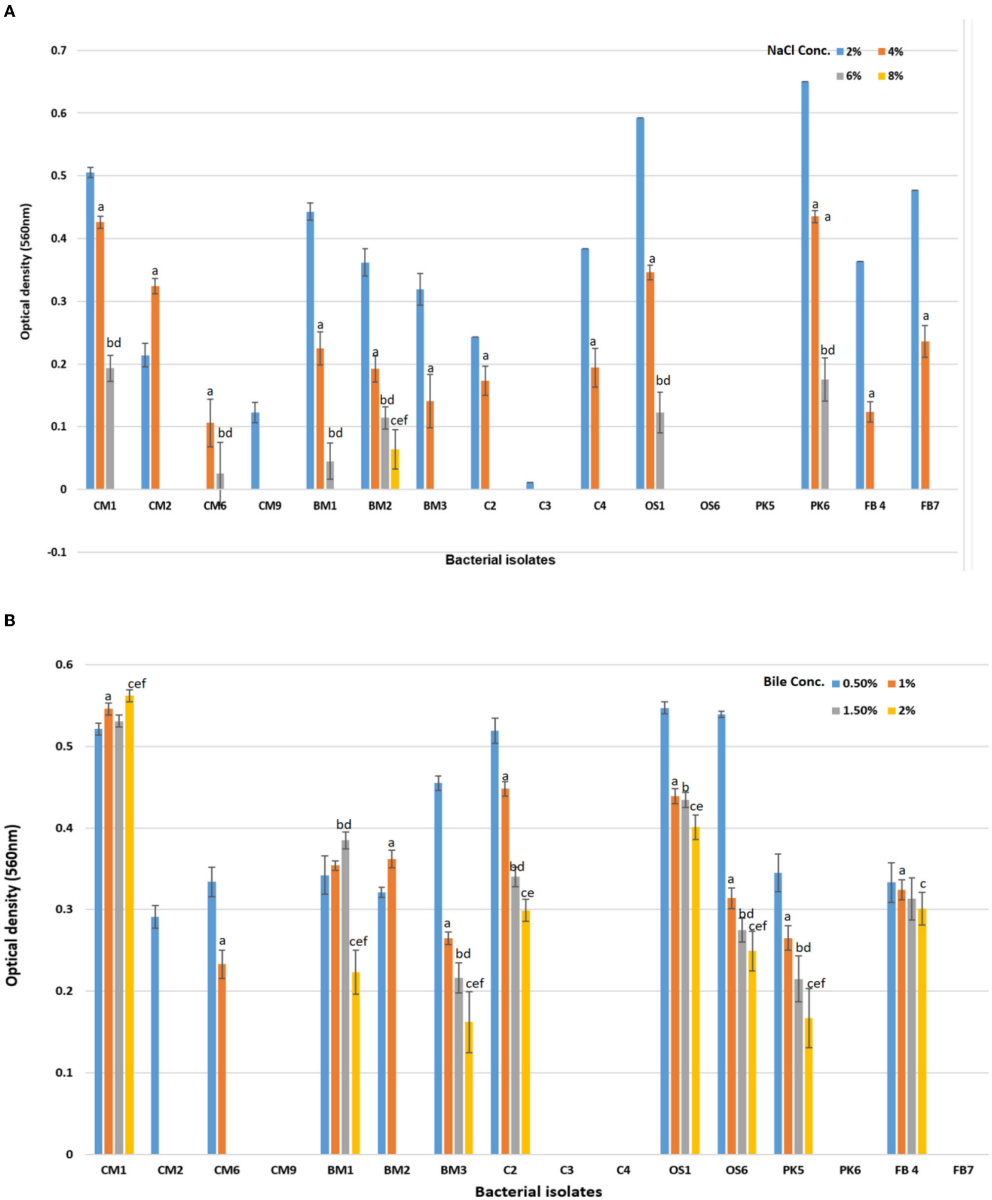
Effect of **(A)** NaCl % concentration (2, 4, 6, and 8%) on the survival rate or growth of bacterial isolates after 24 h of incubation. Superscript small case alphabets presented in chart bars are presenting the significant difference at *p* < 0.05 between trials (% NaCl 2–4: a, 2–6: b, 2–8: c, 4–6: d, 4–8: e, 6–8: f). **(B)** Effect of Bile salt concentration (0.5, 1, 1.5, and 2%) on the survival rate or growth of bacterial isolates after 180 min of incubation at 37°C. Superscript small case alphabets presented in chart bars are presenting the significant difference at *p* < 0.05 between trials Bile conc. 0.5–1: a, 0.5–1.5: b, 0.5–2: c, 1–1.5: d, 1–2: e, 1.5–2: f). *All data were expressed as mean ± SD.

To determine the impact of various NaCl concentrations on growth, a set of experiments was performed and the outcomes are shown in the Figure 2A. The findings indicated that 75% of the isolates demonstrated significant growth at NaCl concentration levels of up to 4% (*p* < 0.05). However, when the concentration was raised to 6 and 8%, only six and one isolate exhibited growth, respectively, as presented in the Figure 2A. Among these results, CM1, OS1, and PK6 demonstrated consistent growth up to NaCl levels of 6%. Values were means of three replicates, dissimilar letters show significant difference (*p* < 0.05).

In a separate study, 16 chosen isolates were subjected to varying levels of bile salts to assess their tolerance. Bile salt tolerance is critical for probiotics to survive in the human gastrointestinal tract (Bazireh et al., 2020). The results, presented in the Figure 2B, demonstrated that eight bacterial isolates (CM1, BM1, BM3, C2, OS1, OS6, PK5, and FB4) demonstrated significantly different degrees of bile salt tolerance up to a concentration of 2% (*p* < 0.05). Four isolates, CM2, CM6, and BM2, survived for a brief period at lower bile salt concentrations (≤1%) but were unable to persist for 24 h at high bile salt concentrations. In contrast, CM9, C3, C4, PK6, and FB7 were unable to survive even at low bile salt concentrations (0.5%). However, CM1, BM1, BM3, C2, OS1, OS6, PK5, and FB4 were found to be able to survive at both low and high concentrations of bile salt, making them beneficial as probiotics (Xing et al., 2016). Values were means of three replicates, dissimilar letters show significant difference (*p* < 0.05).

**Figure 3:**
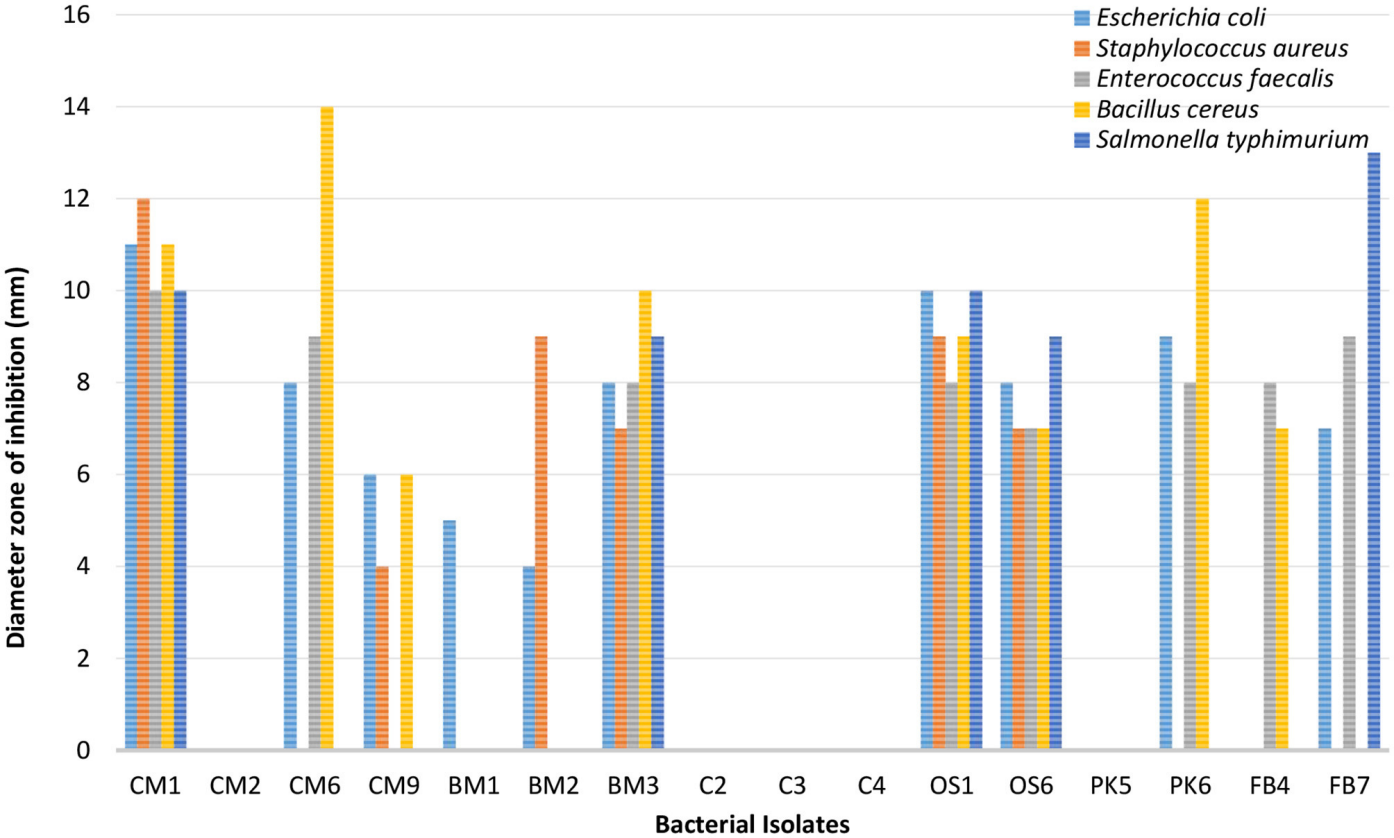
Antimicrobial susceptibility results of bacterial isolates against five enteric-pathogenic bacteria (*E. coli, S. aureus, E. faecalis, Bacillus cereus, S. typhimurium*).

**Figure 4:**
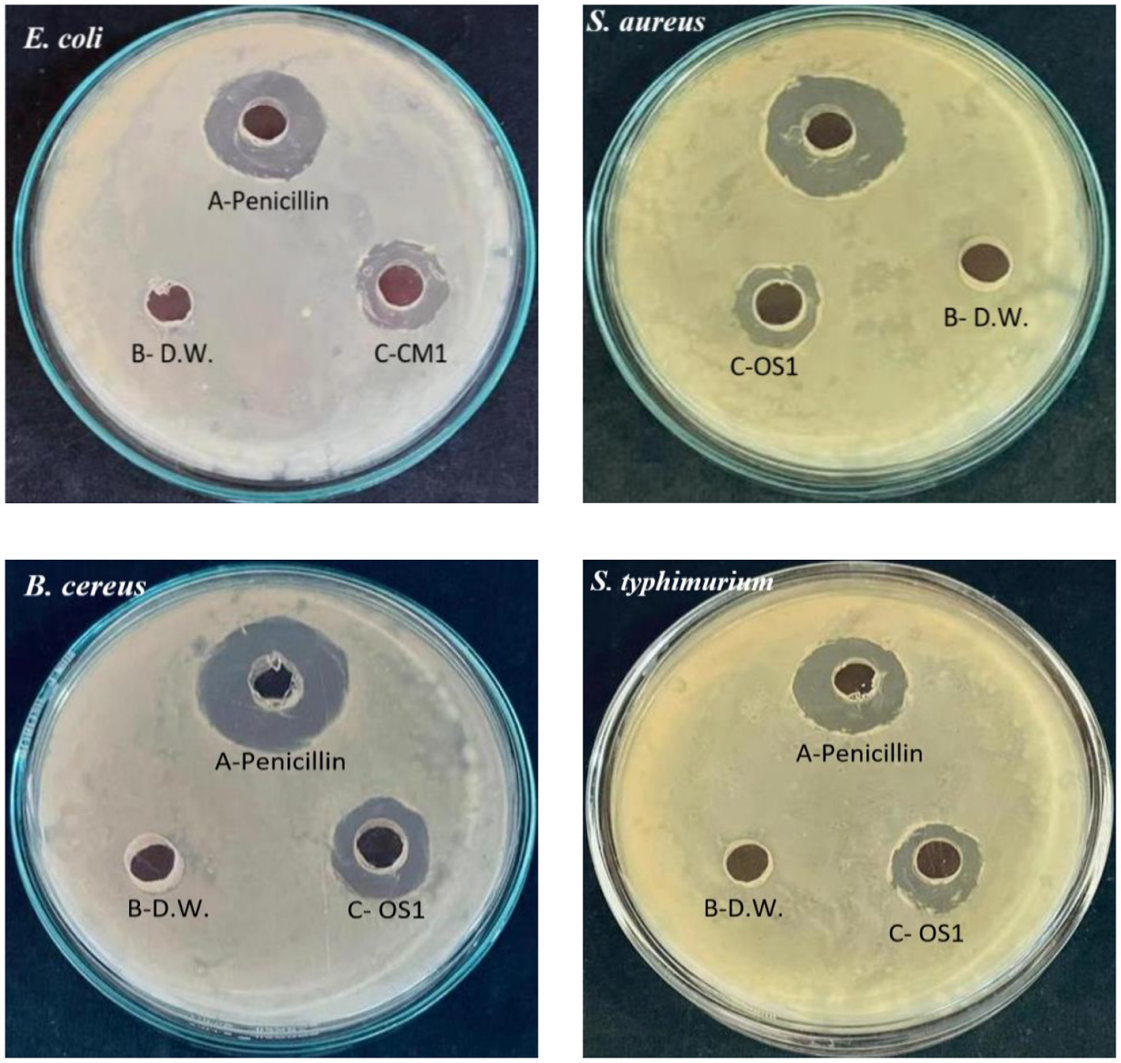
Antimicrobial activity of CM1 and OS1 against enteric pathogens *Escherichia coli, Staphylococcus aureus, Bacillus cereus*, and *Salmonella typhimurium* (A) Positive control, Penicillin; (B) Negative control, Distilled water; (C) Test organism, isolate CM1 or OS1.

In Figures 3, 4, it was observed that isolate CM1 exhibited the highest level of antimicrobial activity with zone of inhibition measurements of 11 mm for *Bacillus Cereus*, 11 mm for *E. coli*, 10 mm for *E. faecalis*, 12 mm for *S*. *aureus*, and 10 mm for *S*. *typhimurium*. The diameter of the zones of inhibition varied between 4 and 14 mm for different isolates against the tested indicator organisms. In contrast, isolates CM2, C2, C3, C4, and PK5 did not exhibit any antimicrobial activity against the tested microorganisms.

The production of primary metabolites such as lactic acid, ethanol, and carbon dioxide, as well as antimicrobial compounds like bacteriocins, are considered to be responsible for the inhibitory properties of LAB species (Wasana et al., 2022). As per the results obtained from assessing temperature, pH, NaCl concentration, and Bile salt tolerance, isolates CM1 and OS1 were selected for further genotypic identification, as they displayed inhibition against all the tested enteric pathogens.

### 3.4. Genotypic characterization

**Figure 5:**
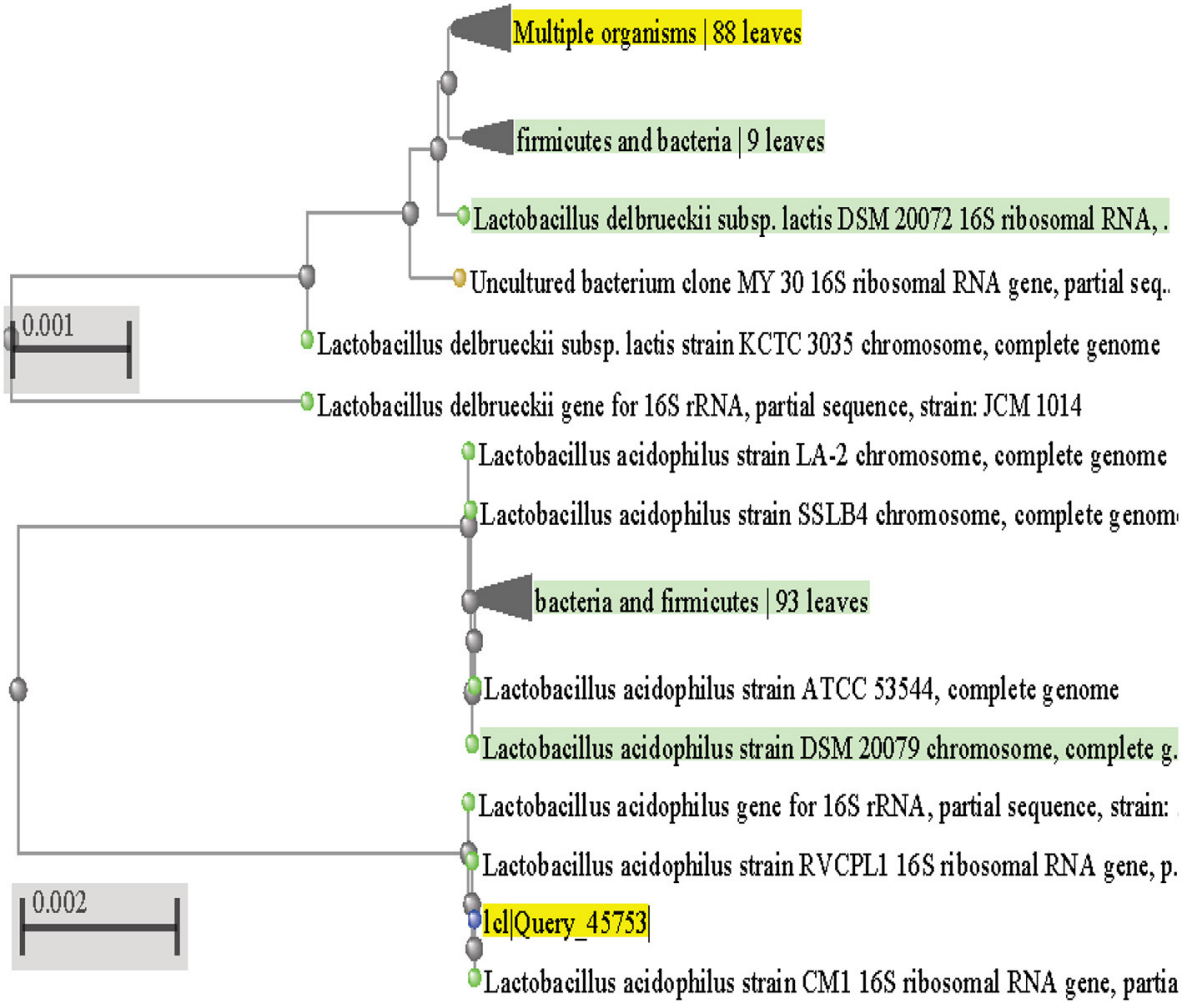
Phylogenetic tree for *L. acidophilus* CM1 and *L. delbrueckii* OS1.

In order to determine the genotypic characteristics of the two isolates CM1 and OS1 which showed the highest potential to be a suitable probiotic, total genomic DNA was extracted from each of these isolates. This extraction was performed in order to identify the isolates through the amplification of the 16S rRNA gene, which is specific to bacteria. The isolates were specifically selected for 16S rRNA gene sequencing as it allows for the identification of the bacteria up to the genus and species level (Sadrani et al., 2014). The gel electrophoresis of the PCR amplicons of CM1 and OS1, both of 1,500 base pair size, was run alongside a marker and successfully demonstrated the successful targeting of the 16S rRNA gene. This result was confirmed through the observation of the electrophoresis gel, which showed the amplicons at the expected size of 1,500 base pairs. After molecular identification, the genomes of both CM1 and OS1 isolates were submitted for phylogenetic analysis and identified as *L. acidophilus* CM1 and *L*. *delbrueckii* OS1 (Figure 5). Latterly sequence data of both isolates were submitted to the NCBI GENBANK in order to obtain their Accession numbers. This was done in order to provide a unique identifier for each of the genomes, which can be used for further research and reference purposes. Overall, the extraction and amplification of the 16S rRNA gene from the two isolates CM1 and OS1 allowed for their identification and ensured their suitability for probiotics through the submission of their genomes to the NCBI GENBANK with accession numbers OP811266 and OP824643, respectively.

## 4. Discussion

Probiotics can also be found in a wide variety of dietary products, the majority of which are dairy products. Many studies (Asadi et al., 2022; Hatami et al., 2022; Rashed et al., 2022) have shown that consuming a probiotic supplement can help with a variety of health issues, including lactose intolerance, hypercholesterolemia, and the prevention and treatment of cardiovascular diseases like atherosclerosis and arteriosclerosis. These health claims are supported by various probiotic products currently offered for purchase in the market. It is possible to improve one's health by including probiotics as part of a diet that is considered to be balanced diet. The ability of microorganisms to raise their host's resistance to disease, colonization potential, antagonize pathogens, and improve overall health are the primary traits that make them promising candidates for use as probiotics (Xing et al., 2016; Al-Dhabi et al., 2020; Salim et al., 2020). In addition to the aforementioned, Abdel-Gawad et al. (2021) mentioned that probiotics could stimulate the immune system and compete with infections for nutrition and adhesion sites. However, the action mechanisms of probiotics are not yet completely known. Despite this, probiotics may demonstrate numerous advantages, potentially integrating bacterial antagonism with effects on the host, like increasing immunity or growth, competitive exclusion, enzyme activation, hormonal suppression, and enhanced immune system response (Al-Dhabi et al., 2020; Lashani et al., 2020; Pumriw et al., 2021; Zhang et al., 2022). Probiotic strains obtained from their native host or consumable food are favored since they are already familiar with the GIT and can flourish and display the intended positive effects more effectively than strains isolated from other sources (Asadi et al., 2022). Consequently, it is crucial to prepare host-specific probiotics to maximize their health effects. In addition, direct examination of putative probiotics *in vivo* can be a time- and resource-intensive and expensive process. As a result, the *in vitro* evaluation is the primary criterion for the screening of probiotics, with the goal being to identify the strain that is the most effective, appropriate, and optimal in terms of its beneficial features (Hatami et al., 2022).

In the current research, particular LAB strains were isolated from the Oral cavity and food samples using MRS medium with a pH range from 6.4 ± 0.2 to 6.5 ± 0.2. These strains were then tested to determine whether or not they had the ability to act as probiotics. Earlier reports reported that a pH range between 6.2 and 8.5 is most suitable for the development and screening of LAB (Anjum et al., 2023). As shown in the Table 2, a total of 60 LAB strains were isolated from the oral (10 strains) and food samples (50 strains), and then analyzed for their microscopic, macroscopic, biochemical, and contemporary *in vitro* probiotic characteristics before being subjected to molecular characterization. We found 60 different strains throughout our study, and of those, 38 were rod-shaped and 22 were cocci. Eighteen of the isolates had a negative reaction to catalase; all were Gram-positive and did not produce spores. Adjoudj et al. (2020) obtained lactic acid bacteria from goats' milk and other fermented foods in the dry zone of Algeria. They found that all of the isolates were Gram-positive, did not form spores, and were negative for catalase. As lactic acid bacteria are obligate anaerobes and flourish without oxygen, Zourari et al. (1992) and Bazireh et al. (2020) reported that these microorganisms do not generate cytochromes or catalase. Lactic acid bacteria are susceptible to oxygen poisoning because hydrogen peroxide is often produced from it, and these bacteria do not have the catalase enzyme needed to destroy it (Zhang et al., 2020). Hydrogen peroxide is decomposed via the following reaction in aerobic organisms due to the enzyme catalase:


$$\begin{array}{l} 2{\text{H}}_{2}{\text{O}}_{2}\to 2{\text{H}}_{2}\text{O}+{\text{O}}_{2} \end{array}$$

The negative catalase results in present study, is a key feature of the isolated probiotic bacteria that show their inability to regulate the breakdown of hydrogen peroxide into water and oxygen. The absence of bubbles during the test indicated the lack of catalase activity, a well-known characteristic of *Lactobacillus* bacteria, as confirmed by Bazireh et al. (2020) study. Lactic acid bacteria are unique in their ability do not to produce the catalase enzyme, which breaks down hydrogen peroxide into water and oxygen. Forouhandeh et al. (2010) isolated lactic acid-forming bacteria from traditional and local cheese and yogurt and tested their biochemical characteristics using various carbon sources. In present study, the fermentation of glucose resulted in the production of acid, not gas, in Durham's tube, and most of the strains were able to ferment glucose (91.63%), lactose (83.3%), and maltose and fructose (66.64%). Other sugars such as mannose (58.31%), galactose (49.98%), mannitol, ribose, sucrose (41.65%), starch (16.66%), and arabinose (8.33%) were fermented to varying degrees. These findings are consistent with Khedid et al. (2009) study, where 25 lactic acid bacteria were isolated from hump-backed camel milk and found to have the ability to ferment glucose, lactose, and maltose. The carbohydrate fermentation method can differ among studies, with some using API kits (Chammas et al., 2006) or eight different types of carbohydrates. Chandok et al. (2014) and Bazireh et al. (2020) used eight different forms of carbohydrates in their study. Khedid et al. (2009) determined the prevalence of LAB using specific criteria, including the ability to produce gas from glucose, methyl red positive, Voges-Proskauer negative, citrate utilization negative, and nitrate reduction positive. The majority of the isolates were methyl red positive due to their ability to ferment glucose into pyruvic acid, which was then metabolized through the mixed acid pathway into various acids such as lactic acid, acetic acid, and formic acid, leading to a decrease in pH below 4.4 and a positive methyl red reaction. However, the Voges-Proskauer test produced negative results, indicating the organisms were unable to produce acetylmethylcarbinol during the digestion of glucose (Khusro et al., 2021). In present study, the H_2_S production test did not result in any darkening of the medium, indicating that the isolates did not possess the ability to convert sulfur compounds to sulfide. It is possible that the sulfide compounds combined with iron compounds to form FeS, which is a dark precipitate, making it difficult for the organisms to reduce the sulfur compounds to sulfide (Chen et al., 2022).

Moreover, the indole production test results indicated that the isolates could not use tryptophanase to produce indole from tryptophan. Kovac's reagent did not cause any change in the surface layer, which remained yellow instead of turning coppery red. Citrate utilization tests revealed that only one isolate, OS1, could use citrate, while the remaining 15 isolates were citrate negative. This finding is consistent with Ayhan et al. (2005) study, which also reported that nine isolates from fermented goat milk were negative for citrate testing. The inability of the isolated bacteria to utilize citrate as their sole source of carbon and energy was also confirmed by the citrate test, which did not result in the formation of a blue color in any of the test tubes. Consequently, the bacteria were unable to produce sodium bicarbonate or ammonia.

### 4.1. Characterization and screening of isolates for probiotic properties

The FAO recommends that probiotics, which are microbial strains that promote health, be safe for their host (Hou et al., 2023). To ensure this safety, it is recommended to select strains that do not have hemolytic activity, indicating their non-virulent nature. In present study examining LAB strains for hemolytic activity, 16 strains were found to be non-hemolytic, making them suitable candidates for use as probiotics. These results are consistent with the findings of previous studies by Asadi et al. (2022), Das et al. (2022), and Wei et al. (2022), who identified various non-hemolytic *Lactobacillus* species isolated from different dietary foods and millet-based alcoholic drinks, and Wang et al. (2018), who found no evidence of probiotic hemolytic activity in lactic acid bacteria from Chinese naturally fermented non-dairy food items. Many researchers (Asadi et al., 2022; Das et al., 2022; Wei et al., 2022; Chino de la Cruz et al., 2023) have reported similar findings, indicating that probiotics do not possess hemolytic activity, which reduce their safety for use as probiotics. In an oxidative/fermentative test, the KIA test is used to determine how bacteria use dextrose. The potential of the lactobacilli to ferment dextrose or lactose, which results in color changes of the pH indicator (phenol) in response to acid generation during the fermentation of the sugars, allows for the distinction of the lactobacilli in this combination. By sticking the butt and streaking the slant with culture for 24 h, KIA media slants were infected. Altarugio et al. (2018) recorded the same results for changes in color of the butt or slant, H_2_S or other gas generation after incubation at 37°C for 18–24 h.

Our study showed that, except BM2, all of the LAB strains tested grown at 37°C after 24 h of incubation. The growth of LAB was limited at both 10 and 42°C. Similar results were found in a previous investigation (Khusro et al., 2021; Wei et al., 2022; Amin et al., 2023), which supports these findings. In addition, all of our LAB isolates showed remarkable salt tolerance, even when exposed to 2% NaCl. However, only six strains shown significantly reduced growth at 6.0% NaCl, and above this concentration, only one strain (BM2) was able to tolerate 10% NaCl level significantly as mentioned in Figure 2A. In a study conducted by Isono et al. (1994), four isolates of lactic acid bacteria could survive at a concentration of 4% NaCl, and one isolate could survive at 6.5% NaCl, both of which were isolated from fermented milk. Similarly, in a study conducted by Khedid et al. (2009), 50% of LAB isolated from camel milk thrived at 40°C, while only 0.5% of the strains were able to develop at 8% NaCl levels. The bacteria in the *Bacillus licheniformis* (BAL) group were highly halophilic, requiring a salt concentration of 5–30% for growth (Hatami et al., 2022; Di Martino et al., 2023). The ability of different genera of lactic acid bacteria to survive on plates containing various NaCl salt concentrations varies. For example, *Leuconostoc, Pediococcus*, and *Lactobacillus* are three genera that can grow and thrive when fermented at high salinity (Bazireh et al., 2020). The results show promising characteristics of prospective LAB probiotics, including the ability to promote bacterial growth and the synthesis of beneficial metabolites. These characteristics of LAB strains are important for the industry, innovation, and long-term preservation. Our study demonstrated that the LAB strains examined grew significantly and optimally at 37°C, and exhibited significant tolerance to 2% NaCl concentration (*p* < 0.05), and had reduced growth at both 10 and 42°C. These findings are consistent with previous reports and highlight the potential of LAB strains as probiotics with desirable traits for industrial and preservation purposes.

Probiotics are beneficial to health for several reasons, including their resistance to antibiotics, bile, and acid in the digestive tract (Xing et al., 2016; Zhang et al., 2020; Yan et al., 2022). Standard *in vitro* investigations are necessary for characterizing putative probiotics to assure their viability and growth in the gastrointestinal system (GIT). Tolerance of intestinal bile salt by prospective probiotic bacteria is a crucial factor for their selection. This is essential to their development and continued existence in the GIT (Amin et al., 2023; Le et al., 2023). In order to colonize their host's gut, probiotics need to be resistant to not just the bile salts but also the acidic environment of the GIT. Most external microorganisms die when they enter the GIT because of the acidic gastric juice secreted, with a pH of about 2.0. For this reason, it is essential that probiotic bacteria can survive in acidic environments (pH 1.0 to pH 3.0) and high bile salt (0.3% w/v) for at least 90 min (Lim and Im, 2009; Kirtzalidou et al., 2011; Xing et al., 2016). In a present study, 16 isolated strains were tested for their survival rates under low pH (pH 1) and high bile salt (>0.3% w/v) conditions for up to 180 min. Ten of these isolates displayed high survival rates (>68%), with significant growth, with strains CM1, BM2, C3, and C4 showing better tolerance and growth at low pH (pH 1) than others. Strain CM2, OS1, and OS6 could bear low pH levels (pH 2). The bilayer membrane structure of these strains is responsible for their high tolerance capability. This structure allows these strains to tolerate negative circumstances, which increases their tolerance capacity easily. However, other studies have found that strains of *Lactobacillus* can resist negative conditions better than strains of other LAB genera (Zhang et al., 2020). These findings support our results. Under acidic conditions, the responses of bile salts on bacterial probiotic cells were shown to be distinct from one another. Moreover, the resistance of probiotics to bile salts was found to be unpredictable and significantly higher than the acid tolerance patterns. Previous investigations (Wang et al., 2023) have indicated that LAB strains isolated from the chicken have a moderate to good survival rate when exposed to a pH 2.0 simulated gastric juice. Similar LAB strains, such as *L*. *pentosus* and *L*. *plantarum*, were identified from fermented sausages, and both flourished in acidic conditions (Cvrtila Fleck et al., 2012). Our study revealed that CM1 and OS1 strains are more tolerant to high bile salt (>1% w/v) and lower pH conditions than other LAB strains and were selected for molecular identification. These findings highlight the importance of characterizing potential probiotic strains to ensure their ability to survive and thrive in the GIT, thereby providing the desired health benefits to their hosts.

### 4.2. Antimicrobial activity

Our study examined the antimicrobial activity of *Lactobacillus acidophilus* CM1 and *Lactobacillus delbrueckii* OS1 strains compared to other isolated bacteria. Our results demonstrated that these strains showed superior antimicrobial activity compared to other bacteria, indicating their potential as probiotics. Our findings were further supported by evidence demonstrating that these strains secrete various compounds, including bacteriocins, biosurfactants, H_2_O_2_, and organic acids, contributing to their anti-microbial activity (Lashani et al., 2020). Additionally, our study revealed that Gram-positive pathogens such as *Staphylococcus aureus, Bacillus cereus*, and *Enterococcus faecalis* were more sensitive to *Lactobacillus acidophilus* CM1 compared to Gram-negative pathogens such as *Escherichia coli* and *Salmonella typhimurium*. However, *Lactobacillus delbrueckii* OS1 demonstrated higher antagonistic activity against Gram-negative bacteria than Gram-positive bacteria. Our findings are consistent with previous studies by Shin et al. (2008); García-Hernández et al. (2016); Taheri et al. (2009); Ayodeji et al. (2017), and Oyewole et al. (2018), which have also reported that LAB isolated from poultry exhibit a wide spectrum of antagonistic activity against various pathogens. These results suggest that *Lactobacillus acidophilus* CM1 and Lactobacillus *delbrueckii* OS1 could potentially be used as probiotics in poultry feed to enhance gut health and protect against pathogenic infections. On the one hand, Kizerwetter-Swida and Binek (2005) observed that LAB strains had higher antagonistic activity against Gram-positive pathogens, such as *Clostridium perfringens* and *Staphylococcus aureus*, than against Gram-negative pathogens like *E. coli* and *Salmonella*. However, de Almeida Júnior et al. (2015) found no relationship between the degree of LAB antagonistic activity and the Gram type of pathogens tested. Spanggaard et al. (2001) previously found that probiotics can hinder the establishment of heterochthonous bacteria in the gastrointestinal tract (GIT) by controlling pathogens, suggesting that using autochthonous microflora as probiotics can make a significant contribution to pathogen control. Jose et al. (2015) observed that LAB strains obtained from animal rumen were more effective than those obtained from dairy sources at preventing the growth of pathogens.

Our research led us to the discovery of two probiotic organisms, both of which were later described and genotypically confirmed to be members of the *Lactobacillus* genus. These native probiotic strains have unique properties that may make them useful in the pharmaceutical, cosmetic, and food sectors. The findings of this research further underline the importance of oral and food as possible sources of new probiotics with desired functional characteristics.

## 5. Conclusion

The study of *Lactobacillus* species and their potential as probiotics has gained significant attention in recent years due to their numerous health benefits. *Lactobacillus* species are commonly found in fermented food sources, including yogurt, kefir, and sourdough, and have been shown to promote digestive health, boost the immune system, and reduce the risk of various diseases. In this study, *Lactobacillus* species were isolated from local fermented and non-fermented food sources, and were tested for their ability to survive in extreme environments such as high salt concentrations and low pH levels. The results showed that the isolated *Lactobacillus* strains were capable of surviving in these harsh conditions, indicating that they are well-suited for survival in the gastrointestinal system.

Furthermore, the isolated *Lactobacillus* strains exhibited probiotic potential for lactose digestion, casein digestion, and sugar fermentation. This makes them suitable for further probiotic beverage production and suggests that they have the potential to provide benefits for digestive health. However, there is still much to be learned about the specific benefits of *Lactobacillus* species as probiotics. While many studies have shown the general benefits of consuming probiotics, more research is required to uncover the specific benefits of different *Lactobacillus* species and their potential as probiotics. For example, further studies could focus on the effect of different *Lactobacillus* species on gut microbiome diversity and the role they play in maintaining a healthy gut. Additionally, studies could also examine the potential of different *Lactobacillus* species to prevent or treat specific diseases, such as inflammatory bowel disease or irritable bowel syndrome. In conclusion, the results of this study demonstrate the potential for *Lactobacillus* species to be used as probiotics, and highlights the importance of further research in this field and can be used for millet based drink production in future study.

## Data availability statement

The datasets presented in this study can be found in online repositories. The names of the repository/repositories and accession number(s) can be found at: https://www.ncbi.nlm.nih.gov/genbank/, OP811266.1; https://www.ncbi.nlm.nih.gov/genbank/, OP824643.1.

## Author contributions

Khushboo: experimental work, data curation, methodology, and writing—original draft. AK: conceptualization, editing of the manuscript, revision, and rewriting. TM: proofreading manuscript and revision and submission of the article. All authors contributed to the article and approved the submitted version.
